# Enhancing COVID-19 Vaccine Acceptance Within Scotland Black, African, and Caribbean Communities and Lessons for Future Vaccination Programmes

**DOI:** 10.1007/s40615-024-02277-6

**Published:** 2025-01-07

**Authors:** J. Adekola, J. G. Audu, T. Okey-Adibe, A. Abubakar, M. Lance, C. Blaize, M. Miragoli

**Affiliations:** 1 Adam Smith Business, School University of Glasgow, 2 Discovery Place, G11 6EY Glasgow, Scotland; 2 Glasgow Caledonian University, Glasgow, Scotland; 3 Central Bank of Nigeria, Lagos, Nigeria; 4 Women in Action Group, London, UK; 5 Unison Glasgow, Glasgow, UK

**Keywords:** COVID-19, Vaccine acceptance, Vaccine programme experience, Black communities, Good health and wellbeing, Reduced inequalities

## Abstract

This study highlights how the intersection of multiple factors shapes the experiences of Scotland’s Black, African, and Caribbean communities in their access and uptake of COVID-19 vaccines in the vaccination programme’s first, second, and booster stages. There was particular interest in understanding the vaccination journey, from scheduling an appointment to attending the appointment. Data in this study was collected between the 1 and 30 April 2022 using a triangulated approach, including a survey (with 408 responses), interviews (26), and focus group discussions (5 groups involving 30 participants). The study shows that 62% of respondents found scheduling a COVID-19 appointment easy, with less than 1% of respondents indicating that the process was complex. Online booking, appointment letters, and walk-in appointments were the most common ways of securing vaccination appointments. Letter appointments, specifically the blue envelope, were beneficial reminder mechanisms. It also provided information about COVID-19 vaccines and what to expect when attending the appointments. Other forms of securing vaccination appointments, such as through GP surgeries, were less commonly used. Around 21.5% of participants felt that receiving an appointment letter provided useful pre-vaccination information and a helpful reminder for their appointment. The accessibility of the vaccination centre, professionalism of the staff, and friendly approach enhanced the vaccine user access, use, and experience of COVID-19 vaccination.

## Introduction

Vaccines are instrumental in mitigating the effects of diseases such as smallpox, polio, and measles [1]. Similarly, COVID-19 vaccines offer a powerful mechanism to scientifically reduce the spread of COVID-19 and the severity of the illness from the virus [2]. Therefore, many national Governments, including the UK and the devolved Government of Scotland, adopted COVID-19 vaccination programmes to manage the pandemic. Vaccination has many benefits: firstly, it protects individuals from the impact of COVID-19 infection, particularly those at higher risk of severe illness [3]. Second, it reduces the burden on the national healthcare systems, including the UK’s National Health Services (NHS), associated with COVID-19 hospitalisations and deaths [4]. Vaccination programmes enable health practitioners to achieve population immunity [5]. Population immunity helps reduce infectious disease transmission diseases [6], [7]. Hence, it is essential to understand the underlying factors driving vaccine inequities in communities.

Individuals’ decision to take (or not) take COVID-19 vaccines navigate a complex myriad of personal, societal, and systemic factors shaping their decisions. Therefore, it is important to understand how these factors intersect to shape decisions at the individual level in their access and use of COVID-19 vaccines. This understanding can also inform the future design of COVID-19 vaccines and other vaccination programmes. For Smith et al. [8], the perception of adverse effects of vaccines shape the uptake of vaccines. Awareness of personal susceptibility, perceived side effects, and effectiveness contribute to low vaccine uptake [9]. For Akan et al. [10], improved risk perception and making vaccination convenient and timely increases vaccine compliance. Accessibility to information is crucial for achieving sufficient infant immunisation coverage, with physicians, parents, and nurses playing a significant role in helping those they care for (e.g. children and patients) understand the vaccine program messages [11]. Workplace colleagues’ opinions, chronic disease, belief in vaccination effectiveness [12], and belief in the ability to prevent flu through natural methods were essential factors negatively or positively influencing vaccination behaviour [13].

Therefore, more needs to be done to know the Black, African, and Caribbean (BAC) communities access, use, and experience of the vaccination programme, in the specific context of Scotland. The focus is on understanding factors that shape the uptake of COVID-19 vaccines. This study uses the terms “Black, African, and Caribbean” to identify a population of Scotland that is ethnically African or Black. The BAC population represents around 1.2% of Scotland’s population. Vaccination uptake as of 23rd November 2021 shows that COVID-19 in African populations is much lower than in White populations. According to the data, the first dose rate was at − 70.9 to 89.7%, the second was at 63.6 to 86.2%, and the third dose or booster was at 55.0 to 87.3%.

This study aims to investigate the experience of BAC communities in their access and use of COVID-19 vaccines between the first, second, and third doses of COVID-19 vaccination. Understanding the experiences and perspectives of BAC communities regarding their access to and use of COVID-19 vaccination can help identify gaps in a way that will make it possible to address potential challenges that may arise in the initial phases of new vaccination programmes during a pandemic. Insight from this study can guide public health authorities in how they effectively tailor future vaccination programmes, including communications strategies, to target population groups. It can also inform policy decisions and interventions to improve vaccine access, address barriers, and encourage acceptance of COVID-19 vaccines within BAC communities.

The experience of vaccine access and use is a multifaceted process [14] and is influenced by the broader determinants associated with place-based challenges that often overlap [15]. Physical distance, limited education, finances, and gender inequity can reduce vaccine uptake in communities (e.g. rural areas). Language barriers, cultural differences, and beliefs are additional challenges migrant communities face [15]. By understanding these dynamics, policy and healthcare practitioners can initiate, design, and implement targeted interventions to enhance vaccine uptake and inequalities to improve general health and wellbeing.

In the context of COVID-19 vaccination, Wong et al. [16] found that vaccination uptake was associated with the potential impact of vaccine hesitancy on jobs, income sources, good health, COVID-19 exposure, knowledge of COVID-19, print material as an information source, and perceived family risk in Hong Kong communities. Viswanath et al. [17] link the perceived risk of COVID-19 vaccine uptake to conservative news outlets, low confidence in scientists, and republican affiliation, reducing the likelihood of vaccination [17]. Perceived infectability was positively linked to COVID-19 vaccination intentions, with perceived behavioural control as the most potent mediator [18]. Public health messaging that clarifies vaccine safety, side effects, and effectiveness can foster vaccine confidence and encourage voluntary vaccination [19]. Simkhada et al. [20] identify nine common barriers to COVID-19 vaccination among Nepali people. These include rumours/misinformation, preference for home remedies, religious restrictions, negative experiences with the influenza vaccine, and worries about side effects. These suggest that there are several factors shaping the uptake of COVID-19 vaccines which requires a multifaceted approach to improve vaccination uptake within targeted communities. Altman et al. [21] argue that embedding COVID-19 vaccination with routine vaccinations increases the chances of children being vaccinated.

Clear communication in addressing vaccine hesitancy [22] and consideration of cultural and linguistic diversity in the communication strategies [23] can positively impact vaccine acceptance. Social media platforms and how they spread misinformation and conspiracy theories can undermine public trust in vaccines and complicate communication efforts [24]. Therefore, providing accurate and easily understandable information about vaccines, safety, and efficacy can help counter misinformation and build public trust [22].

What is also important is the need for healthcare practitioners to continue to engage and communicate with individuals through their vaccine-use journey [25]. Ellingson et al. [26] highlight the importance and the influence of healthcare provider recommendations on vaccine decision-making. Dube et al. [27] argue for a supportive provider-patient interaction to enhance communication and foster vaccine confidence. This means training healthcare practitioners to communicate important vaccine information. This is important due to vaccine misinformation. Vaccine (mis)information is often spread through social network perpetuating doubts and scepticism among individuals [28]. Long term stigma surrounding vaccines within the ACB communities can contribute to vaccine hesitancy and reluctance to vaccinate [28].

The Health Belief Model (HBM) [29] highlights the importance of individual perception and the severity of threats in shaping health behaviours. HBM argues that individual perceived benefits and barriers to mitigating action shape behaviour [30]. The Theory of Planned Behaviour (TPB) [31] also highlights that attitudes, behaviour, societal values, and perceived behavioural control show intention to engage in risk-mitigating behaviours. The Social Ecological Model (SEM) [32] emphasises the interconnectedness of multiple levels of factors at the individual, interpersonal, organisational, and community/societal factors negatively or positively influencing health behaviours [32].

Despite this helpful knowledge, what remains unclear or has received very little scientific attention is the challenges and, specifically, the enablers that shape the vaccine user journey of those willing to take the vaccines. Therefore, one burning question in this research is the following: What factors within a vaccination programme enable or limit a willing individual’s uptake of COVID-19 vaccines?

## Methodology

The University of Glasgow Ethics Committee approved this study. Data was collected using triangulated and mixed-method approach was used to collect the data [33, 34], including the use of a survey, focus group discussion, and one-to-one interviews targeting members of the BAC communities between the 1 and 30 April 2022 following purposive and convenient sampling. The funder, BEMIS, also shared the online survey and advertised the study for interested participants who wished to participate in the interviews. The original report, see (Adekola et al., 2022), submitted to the funder BEMIS analysed key themes from each question. However, this paper re-analysed the data to highlight enabling factors shaping the vaccine user journey and experience of the BAC communities in Scotland, as numerous works focus more on barriers.

We surveyed individuals’ beliefs, attitudes, knowledge, and behaviours related to COVID-19 vaccination. Aside from the participant’s information, we asked 13 questions, including three open-ended questions. A total of 408 responses were received. This provided valuable insights into factors negatively or positively influencing vaccine acceptance or refusal [35].

Regarding the demographics of the respondents, 45.6% and 39.35% were males and females, respectively. 15.2% did not indicate their gender. Regarding age, 75% of the respondents were within the age range of 31–50. Fifteen percent of the respondents were over 50 years of age, and 8.8% of respondents were between 18 and 30 years of age. About 80% of the respondents indicated they were from an African background, and 4.7% and 5.4% indicated they were Caribbean and Black, respectively. The remaining 10% indicated that they were from a mixed background or made no indication. Regarding disability, 88.2 indicated no disability, and 11.8 noted a form of disability. Regarding income, 15.5% earn under £10,000 or no income. 22.3% earn between 11,000 and 30,000, and 17.9% earn above £30,000. 44.35 of the respondents did not indicate their income. Regarding education, 7.1% have a PhD qualification, and 33.8% and 33.6% have an MSc and BSc degree, respectively. 15.9% and 9.3% have a college degree and secondary school certificate. Regarding residential status, 26.7% and 1.2% of respondents have British Citizenship and indefinite leave to remain respectively. 26.5% have work permits, and 11.8% of the respondents are students. 7.6% of the respondents are either refugees or asylum seekers.

Similarly, in the semi-structured one-to-one (26) interviews and five focus groups (30 participants) interviews, 13 open-ended questions also explored participants’ views, experiences, beliefs, and motivations. Focus groups generated further qualitative data using group dynamics [36]. Some participants who completed the survey also participated in the one-to-one interviews. In the one-to-one interview, 56% were females, while 44% were male participants. However, the sample size in the focus group is justifiable as there is substantial health services research literature that recommends very small (3–4 respondents) focus groups for the purpose of reaching more profound and more nuanced understandings of the subject matter [37].

Some participants who completed the survey were invited for one-to-one interviews. Statistical [38] and thematic [39] analyses were used to code the data and key themes raised by participants were identified and categorised into emerging themes presented in the findings section of this paper. However, due to the nature of the collected data (mixed method) and coding the inter-rater reliability can not be established, which is one limitation of the study. Scotland BAC communities are mostly first-generation migrants who come to Scotland, as students, skilled workers, asylum seekers, or refugees. Therefore, they tend to be highly educated.

## Findings

There are key themes that are of interest in this study. This includes the perception and attitude towards the COVID-19 vaccine, factors influencing its uptake, and the impact of the vaccine user journey on vaccine uptake across the various doses of the vaccination programme.

### Vaccine Perception and Attitude

**Fig. 1:**
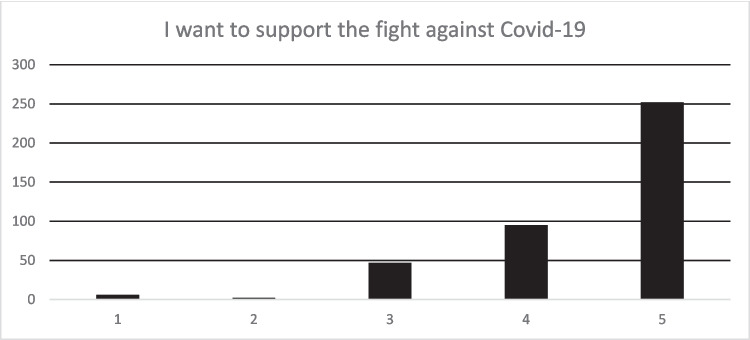
I want to support the fight against COVID-19. Source: study data source (authors own work)

Participants were asked the extent to which they agreed on a scale of one to five—“I want to support the fight against Covid-19”. A total of 408 responses were received. Around 86.3% of the respondents agreed or strongly agreed with the statement. This means there was widespread community support to curb the spread of COVID-19 (see Fig. 1).

In the qualitative data, access to correct information and following COVID-19 guidance were participants highlighted as crucial steps to curb the spread of the virus as reflected in the below quote:

[Individuals] should follow government directives and guidelines regarding C-−19, including social distancing, regular hand washing, use of face coverings and other actions that could help to reduce the spread of the virus. (p.19)

People should get vaccinated, follow personal and environmental hygiene, wash their hands, use face coverings, spread information, enlighten others on the ways mentioned above, and prevent the spread of the virus. Getting tested regularly, especially when you have any of the virus symptoms. Eating healthy food and vegetables is also another way of boosting our immunity. Adhering to the ‘track and trace’ guidelines, mainly when you are contacted that you have been in contact with a carrier of the virus. (p. 27)

Participants were asked several multiple-choice questions to understand their perception of vaccines, including COVID-19 vaccines, regarding their safety, effectiveness, and benefits. Public perception of the need to engage in vaccine trials was of interest in this study. Most (74%) respondents agree that vaccines are safe and should be used as a public health measure. Twenty-six percent either disagreed or were neutral. Regarding public perception of COVID-19 vaccines, 66% believe they are safe and should be used as a public health measure. However, 34% of respondents either disagreed or were neutral.

**Fig. 2:**
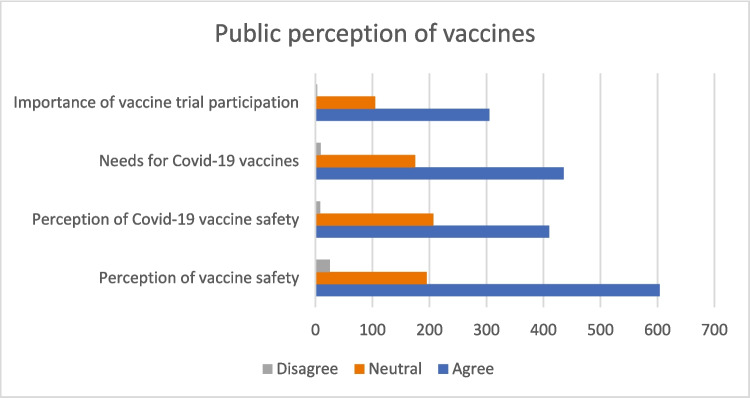
Public perception of vaccines. Source: study data source (authors own work)

Participants were asked of their perceived need for COVID-19 vaccines, 70% of respondents agreed that they are needed to keep everyone safe from the virus. 30% of participants either disagreed or were neutral. Finally, regarding the benefit of the public participating in vaccine trials, 74% agree that it is essential for communities to participate in them. Twenty-six percent of respondents disagreed or were neutral (see Fig. 2).

From the qualitative data, vaccines were seen by some participants as important to public health, including in mitigating virus spreads, enhancing immunity, and reducing the severity of infections, as reflected in the below quote:

The vaccine has assisted a lot in reducing the spread of the COVID-19 virus. While I acknowledge that there are people who do not believe in the vaccine, for me, receiving the vaccine would assist in boosting [my] immune system and not being vulnerable to the virus. (p. 17)

Others worry about the safety and effectiveness of vaccines, especially new vaccines, as reflected in the quote below:

I contracted the virus after receiving the first and second doses of the vaccine. Overall, I do not see the point of the vaccines and how they help to stop the vaccine. (p. 25)

This shows diverse perspectives within the BAC communities regarding vaccines generally and specifically COVID-19 vaccines. It suggests that consistent public health messaging is needed to ensure that people have the correct information to discern between the right information and fake information.

**Fig. 3:**
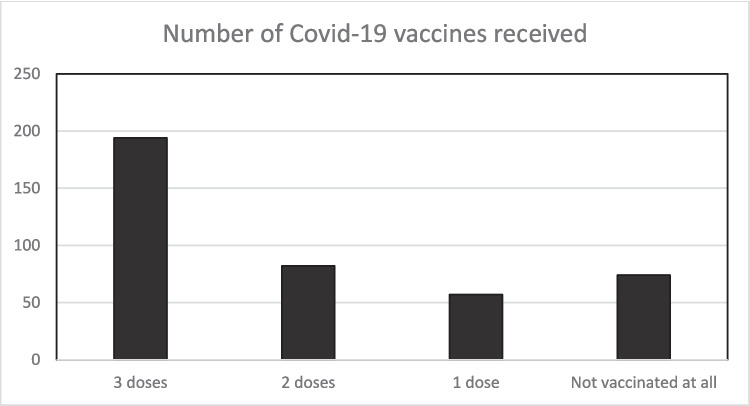
Number of COVID-19 vaccines received. Source: study data source (authors own work)

From the study data, 18.2% of respondents had not taken any COVID-19 vaccines. A total of 14% and 20.1% had received a first and second dose of COVID-19 vaccines, respectively, with less than half of the respondents being fully vaccinated (47.7%) receiving the first, second, and booster doses of COVID-19 vaccine (see Fig. 3). This evidence is similar to that from the data provided by Public Health Scotland earlier in this paper, suggesting that there is some level of hesitancy within the community to take up vaccines.

### Influential Factors in Vaccine Uptake

**Fig. 4:**
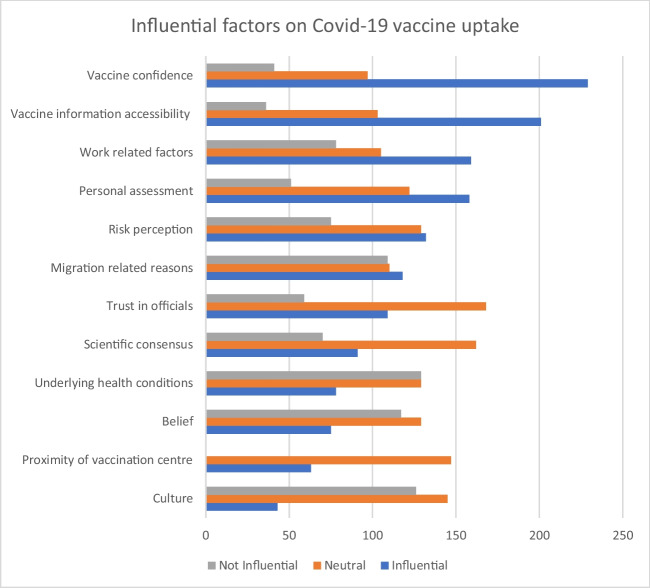
Influential factors shaping COVID-19 vaccine uptake. Source: study data source (authors own work)

Participants were presented with a multiple-choice question about factors influencing their COVID-19 vaccine uptake decisions. Vaccine confidence, information access, work-related factors, personal assessment, and risk perception were highlighted as the most significant factors (see Fig. 4).

The data suggest that having access to the correct information to build vaccine confidence in the recommended vaccines is one of the most important factors in enhancing vaccine uptake within the BAC communities. More specifically, access to the correct information enables members of communities to conduct risk assessments with the right information to develop the right risk perception. In the qualitative study, those who took the vaccine linked it to factors such as information access, work-related factors, personal assessment, risk perception, and social influence, as reflected in the below quotes:

My second and third doses were informed by the fact that I did not react adversely to the first vaccine dose that I received ... I have a very strong conviction in the vaccines. I was also invited by the NHS to receive my vaccines, and I feel it is a responsibility to fulfil my civic duty since I have been invited. (p. 17)

Most of the people I know they have taken all of them. Those people they show it up on social media that they have gotten it so ,most people I know got vaccinated. (p. 2)

What is also important is the ability of people/patients to seek information in accessing the information they need to make the right decisions, as reflected in the quote below:

There [is a] lot of information both for and against the vaccines. One must analyse genuine sources of information from propaganda and conspiracy theories. When certified and official sources provide directives, one should comply. (p. 22).

In this context, guidance from healthcare professionals or vaccinators is needed to increase vaccine confidence among individuals. The data also shows that personal experiences can be essential for vaccine uptake. People who have experienced adverse reactions following vaccination may be less inclined to take further doses of the vaccine. However, seeing friends, family, and peers get vaccinated and receiving positive feedback from them can motivate individuals to do the same.

### Vaccine User Journey of ACB Communities

#### Appointment Scheduling

**Fig. 5:**
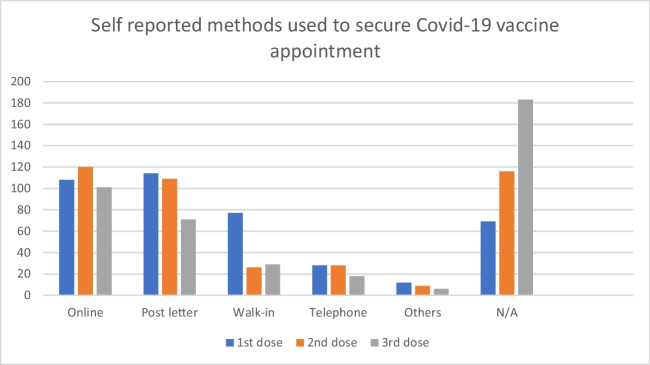
Self-reported methods used to secure COVID-19 vaccine appointment. Source: study data source (authors own work)

Participants were asked to indicate how they scheduled their vaccination appointments. Across the three COVID-19 vaccine appointments received, as indicated in the quantitative study, online booking and letter appointment appointments were used the most to schedule COVID-19 vaccine appointments. Walk-ins at vaccination centres were primarily used in the first dose when compared to the second and third doses. Telephone and appointments received through GP surgeries were less standard in scheduling COVID-19 appointments, as shown in Fig. 5.

In the qualitative study, participants highlighted the importance of official communication channels, such as letters or notifications, in guiding individuals through vaccination. Reliance on transparent communication from healthcare providers remains consistent across experiences, as reflected in the quote below.

I got a letter in through the mailbox for the first one. The second, I confirmed on the App, because I relocated, and I believed I must have missed my letter because of that. The third, I booked online when the Government announced it and I went for it. Overall, the process is very transparent and as long as you are on the NHS record, you will get it. (p. 20)

The quotes above suggest that how members of the public book their vaccination appointments also determine the nature of the information they have before attending a vaccination appointment, which can positively or negatively influence vaccine uptake on the vaccine appointment date. Receiving appointments through letter appointments facilitated access to valuable and relevant vaccine information. The second quote shows that participants use various booking methods to secure vaccine appointments.

#### General Experience in Securing and Attending Vaccine Appointments

**Fig. 6:**
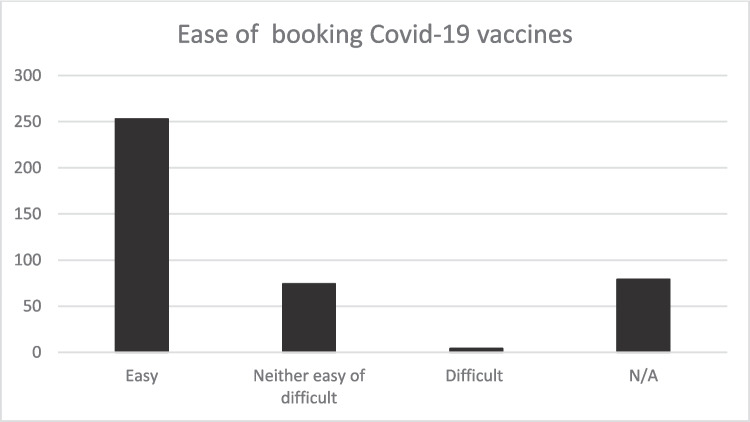
The ease in the process of booking a COVID-19 vaccine appointment. Source: Study data source (authors own work)

Participants were asked to indicate how easy or difficult it was to secure a COVID-19 appointment. A total of 62% of respondents noted that it was easy, and 18.1% expressed that booking an appointment was neither easy nor difficult. 19.4% noted that this did not apply to them as reflected in Fig. 6.

Similarly, the qualitative found that where most of them found the COVID-19 vaccine appointment booking process to be easy, as reflected in the below quotes:

The staff members are very friendly and provide clear information about the vaccine. It took about 15 minutes’ drive from my residence, and I think that’s not far. (p. 25)

Appointment by letter was, ok? The distance was not a problem – the first time I went by taxi and second time I drove there. They explained everything, including side effects and how to mitigate them. They attended to me as professionals should. The timing schedule was ok. (p. 3)

**Fig. 7:**
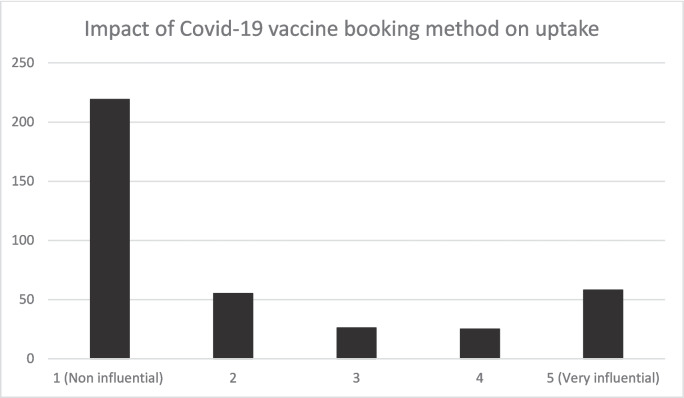
The impact of the COVID-19 vaccine booking method on vaccine uptake. Source: study data source (authors own work)

The themes of convenience, clarity, and staff professionalism emerge as common factors contributing to a positive vaccination experience. From the collected data, 63.6% of respondents did not experience any delay as they were keen to get vaccinated. Nine percent of respondents experienced delays due to factors such as indecision to take or not to take the vaccine, illness, or isolation (Fig. 7).

### Impact of Vaccine User Journey on Vaccine Uptake

Respondents were asked to indicate if how they secured or scheduled their vaccine appointment influenced their decision-making. In total, 385 responses were received for these questions. Seventy-one percent of respondents indicated that how they scheduled their COVID-19 appointment did not influence their decision to take the COVID-19 vaccine. On the contrary, 21.5% felt that how COVID-19 appointments were scheduled influenced their decision to take the COVID-19 vaccine. Specifically, the (letter) method was helpful as it had additional information about COVID-19 vaccines and acted as a reminder (e.g. the blue colour of the envelope) to take the vaccine and of the appointment date and time. Letter appointment was instrumental in shaping people’s expectations of what they would do when attending the vaccine appointment.

According to some of the respondents in the qualitative study:

Yes, the letter was quite instrumental to your taking the first dose. If I had to go online to make an appointment, I wouldn’t have done it. (p.8)

I may not have been vaccinated without a letter of appointment. (p. 9)

Formal invitation by letter appointment acted as reminder and provided people with tangible prompts and access to critical vaccination information. Therefore influenced individuals’ vaccination decisions.

An underlying message here is that the concerted efforts to achieve COVID-19 vaccination equity would require the need to pay attention to logistical, communication, and other social factors. Pursuing COVID-19 vaccination equity means prioritising access to clear information and communication. This way, healthcare providers and policymakers can foster trust and promote widespread vaccine uptake as means of achieving population immunity against the virus.

## Discussion and Recommendations

In this study, four key themes were of interest to explore enabling factors to vaccine uptake in the practical implementation of vaccination programmes: vaccine perception and attitude, influential factors on vaccine uptake, vaccine user journey, and the impact of vaccine use on Scotland’s BAC communities. Data show widespread support from community members to mitigate the spread of COVID-19 and its impact. While 74% believe vaccines generally benefit public health and safety, 66% think this is true for COVID-19 vaccines. This is similar to Subedi et al. [40], who found a high willingness among community members to be vaccinated when invited. Online booking and appointment by letter were the most popular ways of scheduling vaccination appointments. Walk-ins were common in the first phase but less so later. Whereas 74% also believe participating in vaccine trials is crucial, only 18.2% reported not taking any COVID-19 vaccines. The data indicated that about half of the respondents (47.7%) were fully vaccinated, and 14% and 20.1% received one and two doses of COVID-19 vaccines, respectively.

The data indicated that the factors that mainly influenced COVID-19 vaccine uptake were vaccine confidence and information access, work-related factors, personal assessment, and risk perception. These align with Eilers et al. [9], Akan et al. [10], and Swennen et al. [11]. Seventy-one percent of respondents indicated that the methods in which were used to schedule their vaccine appointment were not influential in shaping the decision if they took the vaccine or not. On the contrary, 21.5% found how the appointments were booked as influential. This is similar to Chapman et al. [41], who found that how patients were scheduled to take flu shots impacted the vaccination uptake positively at medical practices compared to only encouraging flu shot takeups.

These findings are necessary for future public vaccination campaigns. Ultimately, an inclusive approach to any vaccination programme will necessitate the involvement of all stakeholders including community members and organisations, experts, and individuals alongside practitioners [42]. Adopting a human rights-based approach to policy development [43] empowers these stakeholders to influence policies that directly impact them. What is also important is the need for continuous efforts at the individualised level to increase awareness of the benefits of vaccines within local communities [44]. It is equally important to emphasise the broader societal advantages of vaccination, especially for vulnerable populations to the public health messaging [45]. While vaccination is essential for individual health, it is also a public health issue so emphasising the social element of vaccine benefit is needed.

It is also essential to provide information concerning different health concerns that may be prevalent in the different communities. For example, there may be a need to provide information regarding the implications of COVID-19 vaccines underlying health conditions affecting the targeted communities [46]. Individuals and groups within communities will have questions and concerns. This means having plans to help them articulate their experiences and manage scientific uncertainties [47]. Door-to-door initiatives, community events, school sessions, and engagement at places of worship are needed to enhance the reach of flexible and targeted vaccination campaigns. This way it is possible to tailor programmes to the needs of specific groups [48, 49]. Recruiting and training community vaccine ambassadors can also help communicate critical health information effectively and dispel myths and misconceptions, particularly those rooted in cultural and religious beliefs [49]. For long-term sustainability, it may be worth embedding vaccination processes into standard healthcare procedures.

It is also essential that practitioners compile a database of community assets within the BAC communities. These assets may include places of worship, local businesses, service providers (such as radio stations), and grassroots organisations. Partnership with community assets will allow for impactful engagement at the community-level. Often, these assets are respected and relied upon by community members [50]. Dispelling myths and conspiracies would also require collaboration with community assets such as places of worship or grassroots community organisations [42]. Combating misinformation involves taking proactive steps, and intervening with credible information and explanations through trusted voices to countering false narratives [51].

Promoting intellectual virtue through education is also essential at the individual level, as individuals are responsible for combatting misinformation [52]. This means encouraging cognitive flexibility training [53] and information search skill development, for example, through the National School Curriculum. Cognitive flexibility training can empower individuals, especially young people, to navigate the digital information landscape effectively. These skills enable critical evaluation of sources, fostering a more informed public capable of discerning credible information from misinformation.

Additionally, the participant data collected in this study suggests that Scotland’s BAC communities have high literacy rates as the study tried to reach different demographics within the community, including refugee and asylum groups, students, and low earners. Around 90% of this study’s participants have completed a college education, and 75% have a BSc qualification. The data also show that at least 7% of the population holds a PhD. However, these high education levels are not reflected in the economic prosperity of community members, as data also show that over 20% of the population earns less than £20,000 per annum or has no source of income. Health authorities must link to the broader determinants of health, such as inequalities in employment and how these might contribute to vaccine inequity, to reduce their social vulnerability to public health challenges.

## Conclusion

Addressing vaccine disparities among different ethnic groups in Scotland would require a multifaceted approach built on community trust-building, targeted interventions, and rigorous research. For this reason, all relevant stakeholders should collaboratively work towards forging solid relationships with trusted community assets, supporting grassroots organisations, and investigating socioeconomic barriers.
